# Biodelivery of therapeutic extracellular vesicles: should mononuclear phagocytes always be feared?

**DOI:** 10.3389/fcell.2023.1211833

**Published:** 2023-07-05

**Authors:** Martyna Cieślik, Krzysztof Bryniarski, Katarzyna Nazimek

**Affiliations:** Department of Immunology, Faculty of Medicine, Jagiellonian University Medical College, Krakow, Poland

**Keywords:** cell-free therapeutics, dendritic cells, exosomes, extracellular vesicles, immunotherapy, macrophages, monocytes, mononuclear phagocyte system

## Abstract

At present, extracellular vesicles (EVs) are considered key candidates for cell-free therapies, including treatment of allergic and autoimmune diseases. However, their therapeutic effectiveness, dependent on proper targeting to the desired cells, is significantly limited due to the reduced bioavailability resulting from their rapid clearance by the cells of the mononuclear phagocyte system (MPS). Thus, developing strategies to avoid EV elimination is essential when applying them in clinical practice. On the other hand, malfunctioning MPS contributes to various immune-related pathologies. Therapeutic reversal of these effects with EVs would be beneficial and could be achieved, for example, by modulating the macrophage phenotype or regulating antigen presentation by dendritic cells. Additionally, intended targeting of EVs to MPS macrophages for replication and repackaging of their molecules into new vesicle subtype can allow for their specific targeting to appropriate populations of acceptor cells. Herein, we briefly discuss the under-explored aspects of the MPS-EV interactions that undoubtedly require further research in order to accelerate the therapeutic use of EVs.

## 1 Therapeutic extracellular vesicles

Extracellular vesicles (EVs), usually divided into exosomes, microvesicles, and less studied apoptotic bodies, are released by all types of human cells and present in all body fluids (Wiklander et al., 2019). However, EV’s isolation, characterization, and classification especially, poses many difficulties, constituting a challenge limiting the practical use of these membranous structures (Théry et al., 2019). In addition, they are isolated from other eukaryotic cells, including fungi (Rizzo et al., 2020) and plants (Urzì et al., 2021), and can also be released by bacteria (Sartorio et al., 2021). The therapeutic potential of these lipid membrane-enclosed vesicles and thus the future development of a new class of EV-based therapeutics has been clearly emphasized in recent years (Conlan et al., 2017; Bernardi and Balbi, 2020; Jahromi and Fuhrmann, 2021; Cheng and Hill, 2022). EVs derived from immune cells, such as T cells, dendritic cells (DCs) or macrophages, as well as from other sources, such as mesenchymal stem cells (MSCs), have a clear immunomodulatory capacity (Zhang et al., 2014; Zhou et al., 2020; Hazrati et al., 2022) due to the expression of costimulatory molecules, antigen presenting activity and transfer of specific cargos, which makes them useful tools in the propagation of anti-tumor response or autoimmune suppression (Marar et al., 2021). New opportunities for EVs’ engineering are proposed for the treatment of neurological, bone, cardiac and metabolic diseases, as well as cancers (Nazimek and Bryniarski, 2020b; Liu et al., 2022; Sun et al., 2023), and in regenerative medicine (Lelek and Zuba-Surma, 2020; Lee and Kim, 2021; Karnas et al., 2023). Moreover, modified EVs are described as promising vehicles for targeted drug delivery, especially in cancer therapy (Chen J. et al., 2022; Sun et al., 2022, 2023; Tan et al., 2022).

## 2 Biodistribution of EVs in the context of their therapeutic efficacy and clearance

*In vivo* biodistribution studies are one of the necessary steps towards the translational application of EVs (De Sousa et al., 2023). Biodistribution of EVs depends on various parameters, including the route of administration, source of parental cells, target cells, as well as the size of vesicles (Wiklander et al., 2015; Murphy et al., 2019). Obviously, the appropriate dose of administered EVs is equally important for their future fate in the organism (Gupta et al., 2021). EVs with their cargos are able to reach different distant organs following various routes of administration, and the most commonly described targeted organs are those enriched in cells of the mononuclear phagocyte system (MPS), and include liver, spleen, kidneys, lungs, intestines, heart and brain (Wen et al., 2016; Manca et al., 2018; Kang et al., 2021; Samuel et al., 2021; Verweij et al., 2021; Driedonks et al., 2022; López de las Hazas et al., 2022; Lorca et al., 2022). These findings suggest the crucial role of MPS cells in the uptake and clearance of exogenously-delivered EVs. Due to the technical difficulties encountered, the issue of MPS uptake of endogenous EVs remains open. However, it can be concluded that the vast majority of vesicles secreted by body cells are naturally removed from the extracellular space by this route. Similarly to other new therapeutics, EVs’ biodistribution studies focus on validation of pharmacokinetic parameters, including half-lives of distribution and elimination phases (Kang et al., 2021). The rapid clearance of therapeutic EVs, resulting in their short half-life in circulation, is one of the main difficulties when adapting them to therapy (Esmaeili et al., 2022; Lu et al., 2022). Accordingly, some researchers point to the relatively short half-life of EV in various tissues, estimated at 30 min or less (Lai et al., 2014; Ronquist, 2019), while others note the time-dependent changes in the circulation and biodistribution of administered EVs, as recently analyzed by Kang et al. (2021).

Under physiological and disease-associated conditions, excretion of EVs into urine or even exhaled air makes them promising biomarkers, but accelerates their removal from circulation (Lai et al., 2014; Lucchetti et al., 2021). However, the crucial role in EVs’ rapid clearance has been attributed to mononuclear phagocyte system.

## 3 Mononuclear phagocyte system

**FIGURE 1:**
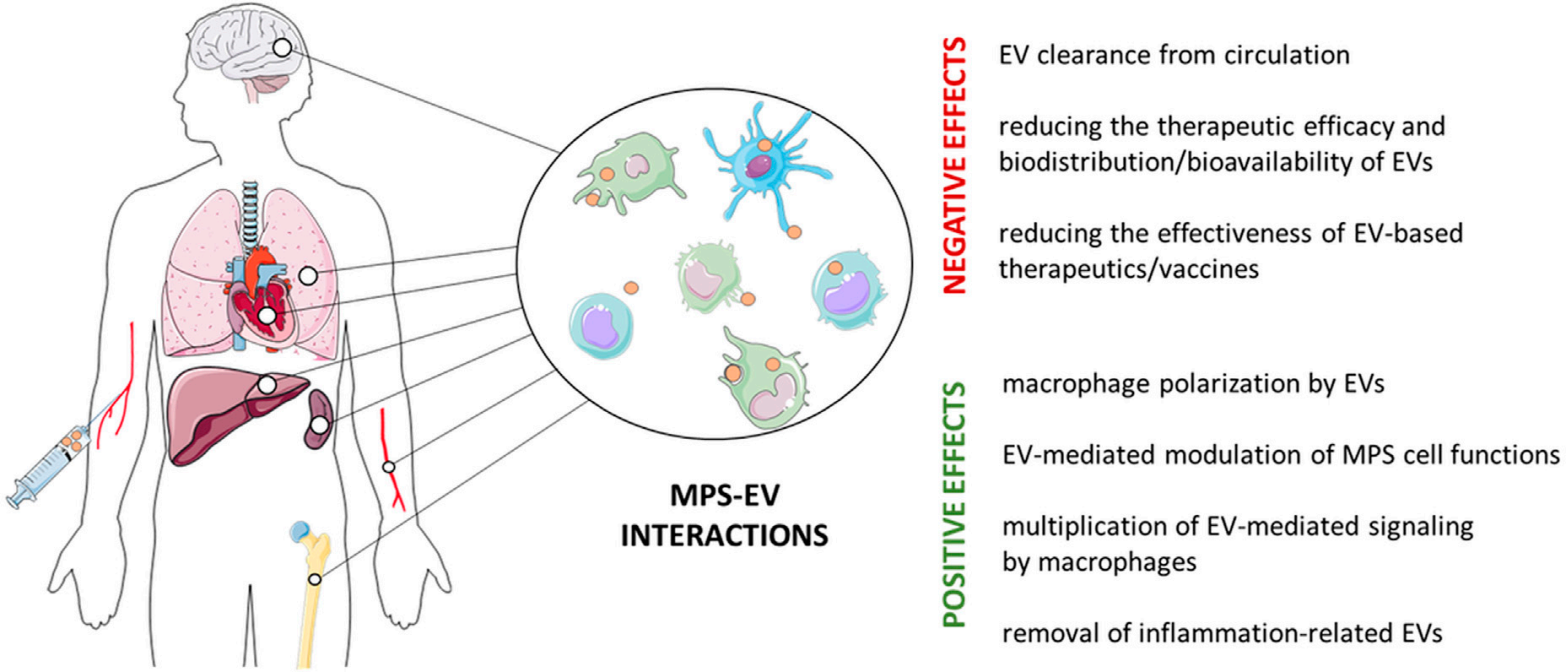
Positive and negative effects of the interaction of the mononuclear phagocyte system (MPS) and extracellular vesicles (EVs). MPS cells, such as monocytes, macrophages and dendritic cells, are found in virtually all body tissues, where they play an important role in tissue homeostasis and in immune defense. However, MPS cells remove both intrinsically-released and therapeutically-administered EVs, which limits their bioavailability. On the other hand, EVs targeting MPS cells can restore their impaired functions to induce the expected biological/clinical effect.

There have been many milestones along the way to the current definition of the mononuclear phagocyte system (MPS), formerly known as the reticuloendothelial system (RES) (Yona and Gordon, 2015). Gordon and Plüddemann (2019) define it as a dispersed organ (Figure 1), due to different tissue residence of MPS cells that include monocytes, macrophages and DCs (Chow et al., 2011), but some authors prefer to focus only on monocytes and macrophages (Hume et al., 2019). MPS cells inhabit all tissues of the body where they can acquire specific, tissue-oriented functions, as in the case of microglia and osteoclasts (Ngo et al., 2022). Conversely, DCs are more motile than macrophages and therefore more likely to migrate to local lymphoid tissues to present antigens during the induction phase of an immune response, while macrophages rather induce an effector phase at the site of inflammation (Hull et al., 2014).

This system is essential for maintaining homeostasis as a major part of the first line of defense against pathogens. Physiologically, MPS is mainly responsible for phagocytosis of self- and foreign antigens, as well as antigen processing and presentation to T cells. Therefore, MPS is considered to link the innate and adaptive immunity (Pahari et al., 2018; Uribe-Querol and Rosales, 2020), and to play a critical role in tissue repair (Viola et al., 2019) as well as in the clearance of damaged, senescent, dying and apoptotic cells (Gordon and Plüddemann, 2018). However, MPS also contributes to immune-related pathologies, especially in infections and chronic inflammation (Hume et al., 2021).

After systemic administration, accumulation of EVs in MPS cell-enriched organs, such as spleen and liver, causes their rapid clearance and inhibit their delivery to distant target organs (Charoenviriyakul et al., 2017; Mentkowski et al., 2018; Tian et al., 2018). Additionally, this may significantly affect the effectiveness of EV-based vaccines, e.g., in anti-tumor immunotherapies (Chen W. et al., 2022) (Figure 1). The lack or significant decline of EVs’ elimination from circulation in macrophage-depleted mice confirms the essential role of MPS in this process (Imai et al., 2015; Matsumoto et al., 2020; Warashina et al., 2022). It is worth to note, however, that EVs released by MPS cells, such as monocytes, appear to be less extensively phagocytosed (You et al., 2022).

MPS cells are also considered the major biological barrier limiting the efficacy of systemically administered therapeutic nanomaterials or synthetic nanoparticles (Cong et al., 2022; Mills et al., 2022; Ruan et al., 2022; Lu et al., 2023), especially due to their accumulation in liver macrophages (Ngo et al., 2022), which promotes research into strategies to avoid MPS phagocytosis.

## 4 Evasion from clearance by MPS

EVs’ escape from phagocytosis has been described as one of the strategies delaying their clearance and improving uptake by targeted cells (Esmaeili et al., 2022). Different camouflage approaches are proposed to avoid reducing EVs amount as a result of MPS action after systemic administration (Parada et al., 2021). The “don’t eat me” signal transmitted by CD47 on tumor cells contributes to the inhibition of their phagocytosis by interacting with signal regulatory protein-alpha (SIRPα) displayed by macrophages (Liu Y. et al., 2023). Also, the expression of CD47 or CD24 molecules protecting against phagocytosis on tumor cell-derived EVs has been reported (Altevogt et al., 2020). A similar strategy is also suggested for therapeutic EVs (Belhadj et al., 2020). Kamerkar et al. demonstrated that CD47 expression on fibroblasts-derived EVs limits their clearance by circulating monocytes (Kamerkar et al., 2017). Additionally, Li Y. et al. (2022) showed that overexpression of this molecule on EVs, unlike cells, does not transmit cell death signals. Zhang et al. (2019) constructed artificial chimeric exosomes by integrating membrane proteins from red blood cells (containing surface CD47) and cancer cells into a synthetic phospholipid bilayer, that have anti-tumor activity and the ability to resist phagocytosis. Similarly, Du et al. (2021) showed that CD47-overexpressing EVs loaded with ferroptosis inducer and photosensitizer effectively evade MPS phagocytosis, which improved their bioavailability and delivery to targeted tumor. Moreover, CD47-containing EVs may competitively interact with macrophage-expressed SIRPα to disturb “don’t eat me” signaling, thereby promoting tumor cell phagocytosis (Cheng et al., 2021). Other molecules that could be expressed by EVs to avoid their phagocytosis and extend the half-life are CD31, CD44, or β2-microglobulin (Parada et al., 2021).

Rapid clearance after intravenous administration disturbs targeted EVs delivery to injured heart tissue (Chen et al., 2021), while therapeutic, miRNA-loaded EVs derived from CD47-overexpressing MSCs were present in serum longer than unmodified EVs and preferentially accumulated in the heart of mice with myocardial infarction reperfusion injury (Wei et al., 2021). A two-step strategy of successful EVs’ delivery to myocardium has also been described recently. First, blocking the macrophage-expressed endocytosis gene *CLTC* for the clathrin heavy chain with EV-delivered siRNA was used to impair the phagocytic activity of hepatic and splenic macrophages. Secondly, therapeutic, miR-21a-containing EVs were injected to significantly improve the cardiac function (Wan et al., 2020).

Another method to reduce the clearance of intravenously administered EVs is based on their conjugation with micelles containing polyethylene glycol (PEG) (Kooijmans et al., 2016). In addition, combination of PEG and CD47 expression on engineered lipid nanoparticles greatly increased their anti-HIV activity by escaping from MPS phagocytosis (Zhang et al., 2023). Modern research approaches propose the use of PEGylation to protect EVs from phagocytosis by MPS cells, which may also support the targeted cargo delivery by constructing “smart exosome platforms” (Guo et al., 2021). Moreover, reduction of the amount of negatively charged phosphatidylserine-derived groups on the EVs’ membranes may also suppress their uptake by macrophages (Matsumoto et al., 2017; Esmaeili et al., 2022).

Various strategies are proposed to solve similar problems with the therapeutic administration of synthetic nanoparticles, especially that Wilhelm et al. (2016) estimated the level of their delivery to solid tumors at only 0.7% of the administered dose. These approaches involve either manipulation of nanomaterials by surface coating with protective factors or changing their shape, or inhibiting and depleting MPS cells (Liu et al., 2017; Ai et al., 2018; Xia et al., 2019; Mills et al., 2022; Lu et al., 2023), and should be combined to increase the biological efficacy. It might be a good idea to use bacteria as an example, as they develop different mechanisms to escape phagocytosis, allowing them to expand and weaken the host’s immune system (Leseigneur et al., 2020; Pidwill et al., 2023).

## 5 Phagocytosis of EVs as a desirable process

Therapeutic functions of EVs depend on the suitable targeting of acceptor cells by direct interaction with extracellular receptors or fusion with cell membrane (Gurung et al., 2021). They are then captured by target cell through different pathways, including caveola-, clathrin- or receptor-mediated and lipid raft-dependent endocytosis as well as macro- and micropinocytosis (Kwok et al., 2021; Pedrioli and Paganetti, 2021; Hazrati et al., 2022). Moreover, internalization of EVs by phagocytosis is also considered (Tkach and Théry, 2016; Jadli et al., 2020). Some studies described phagocytosis as the most efficient mechanism of internalization of cancer and leukemic cell-derived EVs (Feng et al., 2010; Emam et al., 2018). However, EV phagocytosis appears to be a very complex process. Accordingly, observations by Montecalvo et al. (2012) on EV-shuttled miRNA transfer between DCs suggest that EVs release their content to targeted cell cytosol by the complete fusion with the phagosome membrane. This can be preceded by EV hemifusion with the cell membrane followed by endocytosis and/or by internalization as free vesicles.

Moreover, resident alveolar macrophages internalize most of the microvesicles released into the alveoli under the homeostatic conditions (Soni et al., 2022), and their phagocytosis results in alleviation of inflammation during acute lung injury in mice (Mohning et al., 2018), while impaired EV phagocytosis in cystic fibrosis significantly reduces antibacterial immune defenses (Koeppen et al., 2021). The diversity of surface receptors on phagocytic cells allows for the binding of a large number of ligands on the EV surface, which makes phagocytes almost ideal recipient cells (Gonda et al., 2020). Thus, under certain circumstances, it can be assumed that targeting EVs to phagocytes is a desirable process (Figure 1).

## 6 MPS cells as the target of EVs

Under certain circumstances, MPS cells contribute to immune-related pathologies. Thus, targeting them by EVs becomes an interesting immunotherapeutic approach. While maturation, migration, and antigen-presentation processes are the primary targets of DC-directed immunomodulatory EVs (Liu X. et al., 2023), switching and balancing the activation/polarization status appears to be most effective in targeting macrophages (Hu et al., 2021).

As recently reviewed, MSC-derived EVs rather downregulate the antigen-presenting capabilities of DCs (Liu X. et al., 2023), while EVs from other cell sources, including engineered CAR-T lymphocytes, can stimulate the presentation of antigens by DCs, e.g., in cancer (Buzas, 2023).

However, tissue-resident macrophages seem to attract more research attention. Activated microglia are involved in neuroinflammation and related disorders, including neurodegenerations such as Alzheimer’s and Parkinson’s diseases (Muzio et al., 2021). Thus, microglia as MPS cell population can be considered as an interesting target for therapeutic EVs (Xin et al., 2021). Recent studies indicate the possibility of modulating microglial cells by administering EVs isolated from human induced pluripotent stem cell-derived neural stem cells. Following EV administration, a dose-dependent decrease in the secretion of tumor necrosis factor-α (TNF-α) and interleukin 1β (IL-1β) was observed, mediating anti-inflammatory effects of EVs on proinflammatory microglia (Upadhya et al., 2022). Furthermore, the latest findings demonstrated a therapeutic effect of intravenously injected Schwann cell-derived EVs on spinal cord injury by suppressing M1- and stimulating M2-polarization of infiltrating macrophages and microglia (Ren et al., 2023). The latter suggest that EV-mediated MPS cell phenotype switching may produce therapeutic effects.

Accordingly, the contribution of EVs to macrophage polarization and induction of regulatory phenotype is emphasized (Hyvärinen et al., 2018; Li et al., 2021; Gharavi et al., 2022). For instance, MSC-derived EVs reduced IL-23 and IL-22 secretion by CD80^low/intermediate^, CD86^+^, CD163^low^, and CD206^low^ regulatory macrophages, enhancing their anti-inflammatory and tolerance-promoting phenotype (Hyvärinen et al., 2018). Moreover, MSC-derived EVs may polarize human macrophages into radioprotective cells that exhibit high phagocytic activity and have an ability to improve hematopoiesis in mice with lethal acute radiation syndrome (Kink et al., 2019).

In addition, MSC-derived EVs were shown to attenuate myocardial ischemia-reperfusion injury by promoting macrophage polarization towards M2 phenotype (Zhao et al., 2019; Li Q. et al., 2022). Interestingly, fusion of MSC-derived EVs with platelet membrane fragments promoted their trafficking to the ischemic myocardium due to the binding to circulating monocytes (Li Q. et al., 2022). Similar therapeutic effect was observed in myocardial infarction under the activity of miRNA-transferring, DC-derived EVs that activated M2 macrophages in a Treg cell-dependent manner (Zhang et al., 2021).

After local administration, MSC-derived EVs containing therapeutic miRNAs may promote M2 macrophage-mediated angiogenesis and tendon regeneration after its rapture (Xu et al., 2023). Additionally, EVs isolated from adipose tissue-derived MSCs ameliorated tendinopathy by promoting phagocytosis and M2 polarization of macrophages (Wu et al., 2023). Furthermore, stimulating M2 macrophage phenotype by MSC-derived EVs may also improve ligament healing (Chamberlain et al., 2021). Adipose tissue macrophages from lean mice release EVs that modulate macrophage polarization via contained miRNAs to promote wound healing in diabetic mice (Xia et al., 2023), whereas human serum-derived EVs encouraged angiogenesis and osteogenesis by reducing the expression of M1-related genes in macrophages (Xiang et al., 2023). Therapeutic EVs may also diminish the activity of M1 macrophages to alleviate periodontitis (Luo et al., 2023).

M1 macrophages exert anti-tumor activity in cancer environment, and could be induced by miRNA-33- and miRNA-130-overexpressing EVs (Moradi-Chaleshtori et al., 2021) as well as by macrophage-derived EVs expressing human glycyl-tRNA synthetase-1 that trigger cancer cell death (Park et al., 2022). Interestingly, EVs isolated from plasma of post-irradiated patients with cervical cancer promoted the M1 phenotype switch in tumor-associated macrophages (Ren et al., 2022). Similar reprogramming could be induced by tumor cell-derived microparticles loaded with chemotherapeutic drugs (Wei et al., 2023). Furthermore, engineered hybrid cell membrane nanovesicles containing M2-to-M1 repolarization signals and expressing SIRPα prevented both local cancer recurrence and distant metastasis, through triggering an anti-tumor immune response (Rao et al., 2020).

Macrophage activation status in bacterial-host communication may be modulated by EVs. MCS-derived, miRNA-466-containing EVs may participate in the host immune response to multidrug-resistant bacteria by promoting macrophage phagocytosis (Shi et al., 2021). However, internalization of bacterial EVs by macrophages modifies their antimicrobial activity against *Escherichia coli* (Guangzhang et al., 2023). Moreover, both bacterial EVs and EVs derived from infected macrophages may alter macrophage polarization during infection (Qu et al., 2022). Interestingly, microvesicles released by host cells and carrying bacterial pore-forming toxins can be delivered to macrophages, which induces their polarization into the CD14^+^MHCII^low^CD86^low^ cells that exhibit an enhanced response to Gram-positive bacterial ligands (Köffel et al., 2018). Recently, the mechanism of inflammasome activation or silencing in monocytes by EVs isolated from amniotic fluid during pregnancy has been described (Nunzi et al., 2023), suggesting that monocyte activation status may also be modulated by EVs.

Efficient clearance of dying and apoptotic cells by MPS allows for the maintenance of immune homeostasis and peripheral tolerance (Trahtemberg and Mevorach, 2017; Gordon and Plüddemann, 2018). Thus, EV-mediated strategies to restore and/or increase the phagocytosis of apoptotic cells by MPS may induce therapeutic effects in autoimmune and inflammatory diseases. Recently, significantly enhanced efferocytosis of apoptotic cardiomyocytes by macrophages was observed after treatment with EVs secreted by cardiosphere-derived cells to induce the cardioprotective effects (de Couto et al., 2019). Moreover, opsonization of apoptotic cardiomyocytes with MSC-derived EVs significantly increased their phagocytosis by macrophages, which augmented cardiac repair and function (Patil et al., 2021). On the other hand, Chen et al. (2019) showed that apoptotic cell-derived EVs increased macrophage production of transforming growth factor (TGF)-β, which in turn enhanced the clearance of dead cells, which led to the alleviation of colitis.

Our recent findings demonstrated that macrophages can multiply the EV-mediated immunoregulatory signaling (Nazimek et al., 2021). After selective engulfment of suppressor T cell-derived, miRNA-150-carrying EVs that depends on the interaction of antibody light chains with antigenic determinants complexed with MHC class II (Bryniarski et al., 2013; Nazimek et al., 2015, 2018, 2019, 2020), macrophages appear to synthesize additional miRNA-150 molecules and then package them into antigen/MHC-expressing EVs, which enables specific targeting of acceptor T cells (Nazimek et al., 2021). Thus, one can speculate that MPS cells can replicate and repackage immunoregulatory and therapeutic molecules derived from primary EVs to then allow the signal to specifically reach the desired target cell via secondary EV transmission.

## 7 Conclusion

EVs are considered key candidates for cell-free therapies, including allergy and autoimmunity treatment (Nazimek and Bryniarski, 2020a; Nazimek and Bryniarski, 2021). However, EV therapeutic efficacy is affected by limited bioavailability due to their rapid clearance by MPS cells. Thus, strategies to avoid vesicle removal by MPS are considered essential to circumvent the limitations associated with their clinical use. On the other hand, dysregulated MPS cell functions contribute to various immune-related pathologies. Thus, restoring MPS cell activity to normal by EV treatment would be beneficial. Finally, the bystander effect of EV removal by MPS cells can be turned positive by considering macrophages as a multiplier of signaling contained in EVs. Hence, all the aspects discussed briefly in this summary (Figure 1), which have not been sufficiently researched so far, are undoubtedly an interesting direction worth further research in order to accelerate the use of EVs in therapy.

However, future research needs to be directed towards standardization of processes for the production and isolation of therapeutic EVs along with the development of strategies allowing EVs to specifically target the desired cells when administered at established doses, routes and schedules.
